# Bioremediation of industrial wastewater heavy metals using solo and consortium *Enterobacter* spp.

**DOI:** 10.1007/s10661-023-11951-x

**Published:** 2023-10-23

**Authors:** Mahmoud Saber Kelany, Mohamed AbdElAziz El-sawy, Ahmed Rabie El-Gendy, Ehab Aly Beltagy

**Affiliations:** Present Address: National Institute of Oceanography and Fisheries (NIOF), Cairo, Egypt

**Keywords:** Bioremediation, Heavy metal, Industrial waste, *Enterobacter* spp., Suez Gulf

## Abstract

Heavy metals are considered the most common pollutants in industrial wastewater areas. Out of thirty bacterial isolates, only 3 isolates sighted the highest metal resistance activity for Zn^+2^, Fe^+2^, Pb^+2^, Co^+2^, Mn^+2^, Ni^+2^, and Cd^+2^. The biochemical and DNA homology identification with similarities 99.58%, 99.79%, and 99.86% of those isolates was identified and deposited in WDCM, respectively, as *Enterobacter kobei* OM144907 SCUF0000311, *Enterobacter cloacae* OM180597 SCUF0000312, and *Enterobacter hormaechei* OM181067 SCUF0000313. The minimum tolerance activity (MIC) of heavy metal concentrations against *E. kobei* and *E. cloacae* was 25, 15, and 15 mmol/l for Ni^+2^, Fe^+2^, and Mn^+2^, respectively, and 10 mmol/l for Zn^+2^, Pb^+2^, Co^+2^, and Cd^+2^, while against *E. hormaechei*, it is 15 mmol/l for Ni^+2^, Fe^+2^, and Mn^+2^ and 10 mmol/l for Zn^+2^, Pb^+2^, Co^+2^, and Cd^+2^. The consortium and solitary application of bacterial isolates towards heavy metal removal at 100%, 200%, and 300% industrial wastewater concentrations were conducted and showed that more than 90% removal of Zn^+2^, Fe^+2^, Pb^+2^, Mn^+2^, Ni^+2^, and Cd^+2^ from a non-concentrated polluted sample (100%) was reported by the three strains. With doubling the polluted sample concentration (200%), the highest removal efficiency for Zn^+2^, Pb^+2^, Mn^+2^, Ni^+2^, and Cd^+2^ was reported by *E. cloacae* as 70. 75, 66, 65, and 57%, respectively. Removal efficiency after increasing the polluted sample concentration to 300% showed that *E. cloacae* removed above 45% of all tested heavy metals except Pb^+2^. Ultimately, *E. cloacae* exposed the highest efficiency with recommendations for heavy metals removal under higher concentrations.

**Supplementary Information:**

The online version contains supplementary material available at 10.1007/s10661-023-11951-x.

## Introduction

Organic and inorganic pollutants that enter the marine environment have the worst impact and possess a main hazard to all environments and universal ecosystems. Heavy metals, in particular, act as the most influencing hazardous waste that could harm living organisms in any ecosystem. Such harmfulness refers to its toxicity, bioaccumulation, non-degradability, and bio-amplification through progressive trophic levels (Ayaz et al., 2020). A variety of techniques have been applied for remediating the heavy metal contaminants such as precipitation and membrane technologies in addition to ion exchange and electrochemical processes and eventually the biological methods (Ilavský et al., 2015). Generally, heavy metals in trace amounts are playing as essential elements in many metabolic activities of living organisms; however, beyond a certain threshold, they become toxic elements for those organisms causing varying diseases and unstable behavior in living organisms and their ecological systems concerning the non-degradable characteristic of such elements (Mustapha & Halimoon, 2015). As an emerging technique for heavy metal bioremediation, biosorption has proved to be an efficient approach from a point of view of simplicity, flexibility, efficiency, and low-cost methodology focusing on binding the heavy metals on cellular surface structures of biomasses such as bacteria, yeast, fungi, and algae (Espinosa-Ortiz et al., 2016; Rahman et al., 2019).

Microbes are present in our rounded environment, especially in presence of essential elements for growth, where the pollutants may act as co-factors for bacterial growth within certain thresholds. In a sense of that, industrial waste estuaries are considered a suitable place for adopting the growth of all types of microorganisms with certain limitations. For instance, nickel, iron, cobalt, and zinc, which are the dominant industrial waste, play the growth key factor for many bacterial communities, where they possess the appropriate approach to adopt, uptake, and convert them to its beneficiary target (Figueira et al., 2005). Recently, scientists have tended to use bacteria to remove or reduce heavy metals in water and soil. One of those remarkable bacterial families is Enterobacteriaceae. For instance, *Enterobacter* sp., *Enterobacter* cloacae, and *Enterobacter asburiae* are used for bioremediation of Cu^+2^, Cr^+2^, Pb^+2^, Cd^+2^, and Ni^+2^ from different pollution sites (Banerjee et al., 2015; Bestawy et al., 2013; Paul & Mukherjee, 2016; Rahman et al., 2015).

The degree of heavy metal pollution in terms of accumulation pattern is more determined in sediment than in Seawater, where the sediment grain size gives an estimation of the sources, occurrences, and distributions of heavy metals in coastal and estuarine sediments. On the other side, a variety of natural heavy metal accumulation is often located in marine sediments in the shallow and sheltered zones giving the historical variations and the influencing of human activities input in the marine ecosystem (Alloway, 2012; Guagliardi et al., 2013). The retention of heavy metals in marine sediments is probably organized by the rates of finest fractions accumulation, the organic matter decomposition, and Fe^+2^ and Mn^+2^ concentrations (Dar et al., 2016). Consequently, the aim of the current research paper was (i) sample collection targeting the isolation of highly potential tolerant microbes, (ii) minimum tolerance activity (MIC) of isolates for different metal concentrations, (iii) identification of most potent isolates, (iv) sediment sieve analysis, and (v) evaluation of solo and consortium potential isolates towards heavy metal removal of different concentrations: 100%, 200%, and 300% of drainage wastewater.

## Materials and methods

### Sample description and collection

**Fig. 1:**
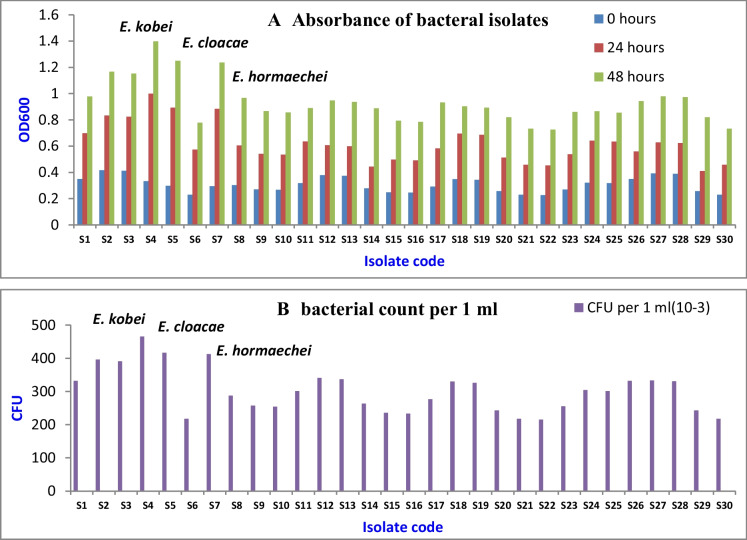
Absorbance of bacterial isolates with colony-forming unit using metals as the supplement of culturing medium

The water and sewage samples were collected under sterilized conditions from different sites of the main industrial estuary drainage in the Adabiya area, Suez, Egypt, in 2021 (supplementary file b figure 1S-a). Samples were aseptically processed for isolation of bacterial spp. using a mineral medium with composition 1 g K_2_HPO_4_, 1 g KH_2_PO_4_, 0.1 g NaCl, 1 g NH_4_NO_3_, 0.5 g MgSO_4_·7H_2_O, 0.1 g Pb (CH3COO)_4_, 0.1 g CuSO_4_, 0.1 g ZnSO_4_, 0.1 g Co(NO_3_)2·6 H_2_O, 10 g yeast extract, 10 g beef extract, and 0.02 g CaCl_2_ in 1 L H2O. The neutral pH level of the prepared medium was adjusted to 7 and incubated for 72 h at 37°C. Supplied chemicals of Sigma Aldrich grad were incorporated in the current research. After incubation, the grown separated bacterial cells were isolated and subcultured using the previous mineral agar medium. To generate the bacterial inoculum for bioremediation, all bacterial isolates were cultivated in a nutrient broth at 37 °C with a shaking speed of 130 rpm for 24 h (Ijoma et al., 2019).

### Heavy metal resistance assessment

The tolerance test depended on the bacterial growth with and without lead acetate, copper sulfate, zinc sulfate, and cobalt nitrate as a metal supplement for medium and bacterial isolates. Briefly, the 30 bacterial isolates were incubated in nutrient broth, and then each isolate was inoculated in five separate flasks. The first flask did not contain any metal supplement with medium and other flasks contained lead acetate, copper sulfate, zinc sulfate, and cobalt nitrate by 1 mM concentration with medium, respectively (Muñoz et al., 2012). Bacterial cell growth for all flasks was determined by measuring OD at 600 nm and microbial counts as colony-forming units (CFU/mL) by serial dilution method (Verma & Kuila, 2019). On the other hand, the agar diffusion method was used to determine the resistance of bacterial isolates to different heavy metals. Well, diffusion plates were prepared using sterile cork borer with poured nutrient agar plates inoculated with overnight cultures of target strains, where 200 microns μm (200 μl) of known concentration (10mmol/l) of tested heavy metals solutions were added in each well, and the plates were incubated at 37 °C for 24 h. After the incubation period, the developed inhibition zone was measured. The lowest clear zone sizes are scored as heavy metal-resistant strains (Kelany et al., 2019).

### Minimum tolerance concentration of bacterial isolates

The highest growth bacterial isolates with different metals were chosen for the determination of the minimum inhibition concentration required for Zn^2+^, Fe^2+^, Pb^2+^, Co^2+^, Mn^2+^, Ni^2+^, and Cd^2+^ remediation. The resistance was determined by the metal dilution method at a concentration of 0.1 to 35 mM. After the addition of the most potent bacterial isolates in Muller–Hinton agar, the plates were pored and inoculated with different metal concentrations by three replicates, and controls without metals were used. Three-day incubation period at 37 °C was proposed for cultivation. The minimum inhibitory concentration (MIC) is defined as the minimum concentration of the heavy metal solution that prevents the growth of bacterial isolates (Gupta Mahendra et al., 2014).

### Identification and characterization of most potent isolates

The most potent isolates were identified biochemically and genetically. The biochemical level was designed by microscopic examination (Ibrahim et al., 2021). The biochemical tests were beta-galactosidase test (ONPG) for lactose fermentation as a tool to differentiate the members of the Enterobacteriaceae, lysine decarboxylase, citrate utilization, hydrogen sulfide production, urease, arginine dihydrolase, tryptophan deaminase, oxidase, ornithine decarboxylase, indole, and Voges–Proskauer. On the other hand, testing different enzyme productions (arabinose, rhamnose, gelatinase, glucose, sorbitol, mannitol, inositol, sucrose, and melibiose) was applied.

A glycerol stock of 20% (glycerol/medium) of pure cultures was prepared and kept for the second identification level, which was genetic identification (Mitra et al., 2018). Identification on gene level was processed. According to the protocol supplied with QIAquick kits (Qiagen, Valencia), genomic DNA and PCR product of 16S rDNA fragment were purified and transferred to the next level. The approach of the Bigdye Terminator V3.1 cycle sequencing kit (PerkinElmer) was applied. The resulting sequence was implemented using the Applied Biosystems3130 genetic analyzer (HITACHI, Japan). Accession numbers for identified strains were given with aid of BLAST® analysis (Basic Local Alignment Search Tool) (Kim et al., 2012). The phylogenetic tree was established by the MegAlign module of LasergeneDNAStar version 12.1 (Abed et al., 2020), and phylogenetic analyses were constructed based on maximum likelihood, neighbor-joining, and maximum parsimony in MEGA6 (Tamura et al., 2013). The identified strains were deposited in the world data center of microbiology (WDCM), Suez Canal University Fungarium (SCUF), Egypt.

### Heavy metal assessment after and before bioremediation for water and sediment

Filtration of water samples by a 0.45-m membrane filter was done, and the heavy metals were pre-concentrated and separated from seawater samples by the ammonium pyrrolidine dithiocarbamate (APDC)/methyl isobutyl ketone (MIBK) solvent extraction technique (Eaton et al., 1995; Folk, 1980). Finally, the metals in the organic layer were extracted using 50% HNO3 and collected in a polyethylene bottle to be analyzed by atomic absorption spectrometry (FAAS PerkinElmer model A Analyst 100) for Zn2+, Fe2+, Pb2+, Co2+, Mn2+, Ni2+, and Cd2+. On the other hand, the sediments were dried for 48 h at 60 °C in a thermostatically controlled oven, homogenized with an agate pestle and mortar and sieved using a 63-μm sieve. In a dry Teflon beaker, 0.5 g of fine sediment powder was thoroughly digested at 85 °C with a mixed acid solution containing HNO3:HClO4 (3:1 v/v) according to the method described by Oregioni and Aston (1984). Studied metals were analyzed by FAAS (PerkinElmer model A Analyst 100), and the results were expressed as mg/kg. Each heavy metal was analyzed in three replicates, and the results were presented as mean (Chester et al., 1994; Oregioni & Aston, 1984)

### Sieve analysis with carbonate and organic matter determination

#### Granulometric analysis

To estimate the granulometric analysis; 100 g of each disaggregated day sample was analyzed mechanically by using a standard set of sieves according to Wentworth scale every one phi (Ø) interval. The collected sieve fractions were accurately weighed. The grain size statistical parameters are mean size (MZ), sorting (δI), skewness (SKI), and the kurtosis (KG) according to Folk (1974) and are computed in the BASIC program “GW-BASIC 3.22” (GRSIZE) according to Rząsa and Owczarzak (2015). Varied sized of seven portions were gathered as follows: gravel (Ø-1), very coarse sand (Ø0), coarse sand (Ø1), medium sand (Ø2), fine sand (Ø3), very fine sand (Ø4), and mud (<Ø 4) (Folk, 1980; Rząsa & Owczarzak, 2015).

#### Geochemical analyses

For the geochemical analyses, about 10g of each sample was ground by agate mortar to less than 80 mesh. Studying the geochemical characteristics of the sediment is designed by measuring total carbonate and total organic matter.

### Total carbonate determination

Carbonate matter in terms of CO3% was measured in the target samples. The adjusted weight (1 g) of was thoroughly mixed with 25 ml diluted glacial acetic acid using shaking apparatus overnight. The remained ground samples after incubation were dried, and the difference in weight, before and after incubation, was considered the carbonate content representing as a percentage of the total weight (Dar et al., 2016). The carbonate percentage was calculated upon the next equation:

$$\textrm{C}{\textrm{O}}_3\%=\frac{\textrm{wt}.\textrm{of}\ \textrm{sample}-\textrm{wt}.\kern0.5em \textrm{of}\ \textrm{residue}\ }{\textrm{wt}.\textrm{of}\ \textrm{sample}}\ 100$$

#### Total organic matter content

After 2 h of incubation at 550°C, 1 g of each sample was burned to ash. Eventually, the organic matter constituent of each sediment sample was measured from consecutive weight loss (Brenner & Binford, 1988; Liu et al., 2019). Upon the following equation, total organic matter was measured:

$$\textrm{TOM}\%==\frac{\textrm{wt}.\textrm{of}\ \textrm{sample}-\textrm{wt}.\kern0.5em \textrm{of}\ \textrm{ash}\ }{\textrm{wt}.\textrm{of}\ \textrm{sample}}\ 100$$

### Consortium application for drain sewage bioremediation using bacterial isolates

*E. kobei*, *E. cloacae*, and *E. hormaechei* were used for bioremediation of Zn^2+^, Fe^2+^, Pb^2+^, Co^2+^, Mn^2+^, Ni^2+^, and Cd^2+^ from the water of industrial drainage wastewater by metal concentration 100 %, 200 %, and 300 %. The composition of the medium used was 1000 ml industrial effluent by different concentrations, 1g K_2_HPO_4_, 1 g KH_2_PO_4_, 0.1 g NaCl_2_, 1 g NH_4_NO_3_, 0.5 g MgSO_4_·7H_2_0, 10 g yeast extract, 10 g beef extract, and 0.02 g CaCl_2_. The removal of heavy metals with various concentrations was tested using bacterial isolates, each type separately, and again with three isolates combined for each metal concentration. The prepared flasks were cultivated for 96 h at 37 °C. Bioremediation patterns were measured every 12 h of incubation by absorbance at 600 nm using a Spekol 1900, UV-VIS spectrophotometer, and metal concentration measurement using PerkinElmer A Analyst 100 atomic absorption spectrometer as illustrated in Section 2.6. According to Ijoma et al., 2019, the bacterial isolates were introduced to the MIC test using water from industrial effluent which was replaced by distilled water and added the components of the medium (Ijoma et al., 2019).

### Statistical analysis

Standard deviation (±SD) with probability (*P*<0.05) was calculated for presenting data. The significance of data using the ANOVA test was evaluated by XISTATE (Microsoft, USA) and GraphPad Prism 4 (USA).

## Result

### Isolation and screening of heavy metal-resistant bacterial isolates

**Fig. 2:**
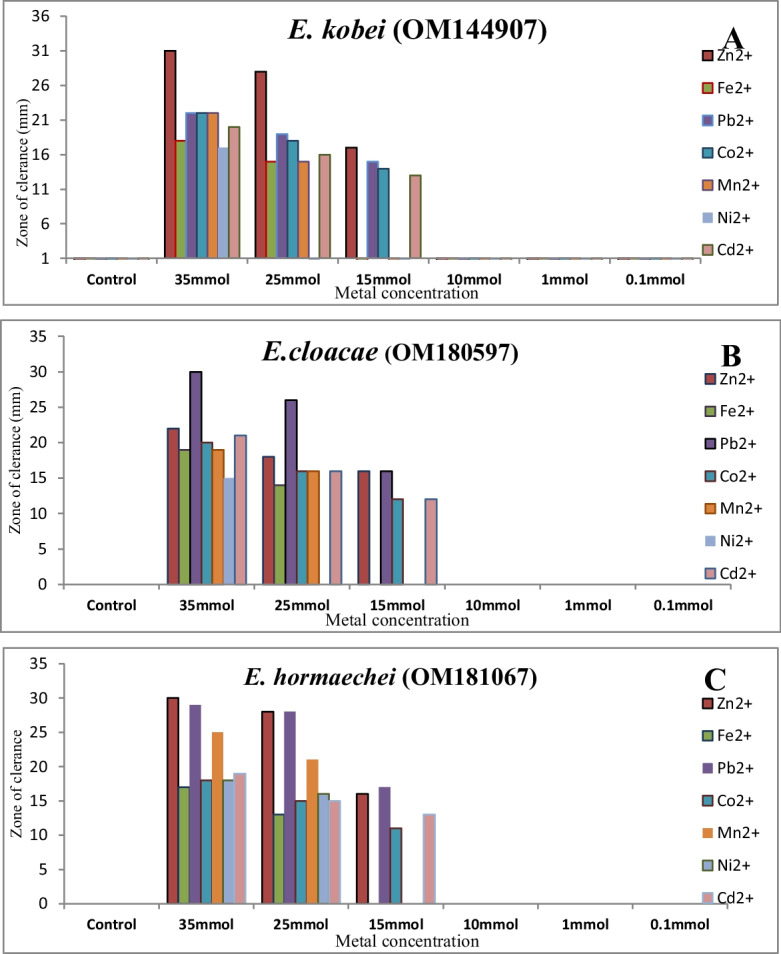
Minimum inhibition concentration of different metal ions for bacterial isolates: **A** *E. Kobei*; **B** *E. cloacae*; **C** *E. hormaechei*

Thirty bacterial isolates were isolated from eight samples of water and sewage that were collected from the main sewage drain in the Al-Adabiya area, Suez, Egypt. All isolated samples were subjected to a growth tolerance test in the presence of different types of heavy metals. After screening, out of these 30 isolates, only 3 bacterial isolates exhibited a varying degree of heavy metal resistance potential against selected heavy metals (Fig. 2a and b). Figure 2 shows the positive and negative resistance of bacterial samples to heavy metals. Table 1 illustrated all isolates’ resistance to the 10 mmol/l concentration of each heavy metal. The most tolerant samples were S4, S5, and S7. Hence, these isolates were selected for further study and identified by PCR sequence analysis.

**Table 1:** Measuring of bacterial tolerance for different heavy metals

	Zone average of clearance (mm) for metals by 10 mmol/l concentration						
	Zn^2+^	Fe^2+^	Pb^2+^	Co^2+^	Mn^2+^	Ni^2+^	Cd^2+^
S1	23±0.46	20±0.6	18±0.72	22±0.33	23±0.92	22±0.66	18±0.45
S2	25±0.5	18±0.54	19±0.76	17±0.26	16±0.64	23±0.69	12±0.3
S3	27±0.54	19±0.57	21±0.84	18±0.27	18±0.72	23±0.69	20±0.5
S4	4±0.08	0±0	7±0.28	0±0	5±0.2	0±0	0±0
S5	5±0.1	0±0	0±0	6±0.1	0±0	4±0.12	5±0.13
S6	23±0.46	16±0.48	15±0.6	18±0.7	19±0.76	18±0.54	14±0.35
S7	0±0	0±0	5±0.2	4±0.06	0±0	5±0.15	6±0.15
S8	14±0.28	16±0.48	17±0.68	18±0.27	19±0.76	21±0.63	16±0.4
S9	18±036	17±0.51	17±0.68	19±0.29	21±0.84	18±0.54	20±0.5
S10	16±0.32	14±0.42	18±0.72	21±0.32	20±0.8	18±0.54	14±0.35
S11	22±044	18±0.54	15±0.6	20±0.3	18±0.72	17±0.51	16±0.4
S12	13±0.26	11±0.33	12±0.48	15±0.23	12±0.48	13±0.39	11±0.28
S13	18±0.36	16±0.48	14±0.56	15±0.23	17±0.68	16±0.48	13±0.33
S14	18±0.36	15±0.45	14±0.56	13±0.2	14±0.56	16±0.48	13±0.33
S15	23±0.46	16±0.48	18±0.72	18±0.27	19±0.76	22±0.66	21±0.53
S16	22±0.44	17±0.51	20±0.8	18±0.27	19±0.76	20±0.6	22±0.55
S17	19±0.38	17±0.51	19±0.76	20±0.3	18±0.72	16±0.48	14±0.35
S18	20±0.4	16±0.48	22±0.88	20±0.3	15±0.6	14±0.42	16±0.4
S19	20±0.4	17±0.51	15±0.6	14±0.21	16±0.64	18±0.54	19±0.48
S20	14±0.28	18±0.54	15±0.6	19±0.29	20±0.8	17±0.51	14±0.35
S21	16±0.32	17±0.51	15±0.6	22±0.33	14±0.56	18±0.54	16±0.4
S22	20±0.4	16±0.48	15±0.6	17±0.26	19±0.76	18±0.54	21±0.53
S23	25±0.5	21±0.63	18±0.72	22±0.33	24±0.96	18±0.54	19±0.48
S24	16±0.32	17±0.51	16±0.64	14±0.21	12±0.48	16±0.48	13±0.33
S25	17±0.34	19±0.57	21±0.84	15±0.23	17±0.68	18±0.54	14±0.35
S26	14±0.28	16±0.48	20±0.8	19±0.26	14±0.56	25±0.75	13±0.33
S27	20±0.4	18±0.54	14±0.56	16±0.24	14±0.56	18±0.54	12±0.3
S28	22±0.44	18±0.54	15±0.6	20±0.3	14±0.56	16±0.48	11±0.28
S29	24±0.48	22±0.66	21±0.84	16±0.4	17±0.68	18±0.54	14±0.35
S30	19±0.38	21±0.63	23±0.92	17±0.26	16±0.64	19±0.57	16±0.4

Figure 1 showed the absorbance and number of bacterial cells in different isolates after 24 and 48 h of incubation. The results showed that samples number four, five, and seven were the growing samples in the presence of heavy metal concentrations. The absorbance of sample number four was 0.3326, 0.9978, and 1.39692 after 0, 24, and 48 h of incubation, and the number of bacteria per ml was 465.64 CFU/ml. Also, the absorbance of sample number five was 0.2976, 0.8928, and 1.24992 after 0, 24, and 48 h of incubation, and the number of bacteria per ml was 416.64 CFU/ml. On the other hand, the absorbance of sample number seven was 0.2944, 0.8832, and 1.23648 after 0, 24, and 48 h of incubation, and the number of bacteria per ml was 412.16 CFU/ml.

Table 1 illustrates the most tolerant bacterial isolates for the presence of 10 mmol of metal ions. Samples four, five, and seven were the most tolerant isolates. Sample four justified the tolerance by zone average of clearance 4, 0, 7, 0, 5, and 0 (mm) for Zn^+2^, Fe^+2^, Pb^+2^, Co^+2^, Mn^+2^, Ni^+2^, and Cd^+2^ metal, respectively. The lowest tolerance rate was demonstrated for Zn2+, Pb2+, and Mn2+, and the highest was for Fe^+2^, Co^+2^, Ni^+2^, and Cd^+2^, respectively. Sample five listed tolerance by 5, 0, 0, 6, 0, 4, and 5 (mm) for Zn^+2^, Fe^+2^, Pb^+2^, Co^+2^, Mn^+2^, Ni^+2^, and Cd^+2^ metal, respectively. The lowest tolerance rate was recorded for Co^+2^, and the highest was for Cu^+2^, Fe^+2^, Pb^+2^, and Mn^+2^, respectively. Finally, sample seven recorded tolerance by 0, 0, 5, 4, 0, 5, and 6 (mm) for Zn^+2^, Fe^+2^, Pb^+2^, Co^+2^, Mn^+2^, Ni^+2^, and Cd^+2^ metal, respectively. The lowest tolerance rate was recorded for Cd^+2^, and the highest was for Zn^+2^, Fe^+2^, and Mn^+2^ metal, respectively.

### Biochemical identification and molecular taxonomy of a selection of heavy metal bioremediation bacterial isolates

Three potent bioremediation isolates (S4, S5, and S7) were extracted and identified using microscopic examination, morphological, biochemically, and biosystems 3130 genetic analyzers. The microscopic examination in supplementary file b figure 2S-b revealed that S4 appeared as a short rod (i), while S5 was a cocci-like structure (ii). S7 showed a typical red shape (iii).

The biochemical tests illustrate differentiation between the three tested potential strains, where ONPG, arginine dihydrolase, lysine decarboxylase, Simmons citrate, tryptophan deaminase, and mannitol played the key elements of differences between those strains; otherwise, all other tests provoked similar results between them. As shown in Table 2, the strain *E. cloacae* expressed positive signs with lysine decarboxylase, ornithine decarboxylase, citrate utilization, H_2_S production, urease, tryptophan deaminase, and Voges–Proskauer test, in addition to positive effect for fermentation of glucose, sorbitol, rhamnose, sucrose, and arabinose. On the other side, ONPG, arginine dihydrolase, citrate utilization, H_2_S, indole, oxidase, gelatinase, mannitol, inositol, and melibiose were negative. *E. kobei* achieved positive reactions for ONPG, arginine dihydrolase, ornithine decarboxylase, citrate utilization, urease, tryptophan deaminase, Voges–Proskauer test, glucose, mannitol, sorbitol, rhamnose, sucrose, and arabinose with the negative reaction for lysine decarboxylase, H_2_S, indole, oxidase, gelatinase, inositol, and melibiose. *E. hormaechei* gave positive reactions for ONPG, arginine dihydrolase, ornithine decarboxylase, citrate utilization, urease, Voges–Proskauer test, glucose, mannitol, sorbitol, rhamnose, sucrose, and arabinose, but the negative reaction was for lysine decarboxylase, H_2_S, tryptophan deaminase, indole, oxidase, gelatinase, inositol, and melibiose.

**Table 2:** Comparison of results in 20 biochemical tests for bacterial isolates

N	Test	*E. cloacae*	*E. kobei*	*E. hormaechei*
1	ONPG	Negative	Positive	Positive
2	Arginine dihydrolase	Negative	Positive	Positive
3	Lysine decarboxylase	Positive	Negative	Negative
4	Ornithine decarboxylase	Positive	Positive	Positive
5	Citrate Simmons	Negative	Positive	Positive
6	H2S	Negative	Negative	Negative
7	Urease	Positive	Positive	Positive
8	Tryptophan deaminase	Positive	Positive	Negative
9	Indole	Negative	Negative	Negative
10	Oxidase	Negative	Negative	Negative
11	Voges–Proskauer	Positive	Positive	Positive
*Enzymes production*				
12	Gelatinase	Negative	Negative	Negative
13	Glucose	Positive	Positive	Positive
14	Mannitol	Negative	Positive	Positive
15	Inositol	Negative	Negative	Negative
16	Sorbitol	Positive	Positive	Positive
17	Rhamnose	Positive	Positive	Positive
18	Sucrose	Positive	Positive	Positive
19	Melibiose	Negative	Negative	Negative
20	Arabinose	Positive	Positive	Positive

**Fig. 3:**
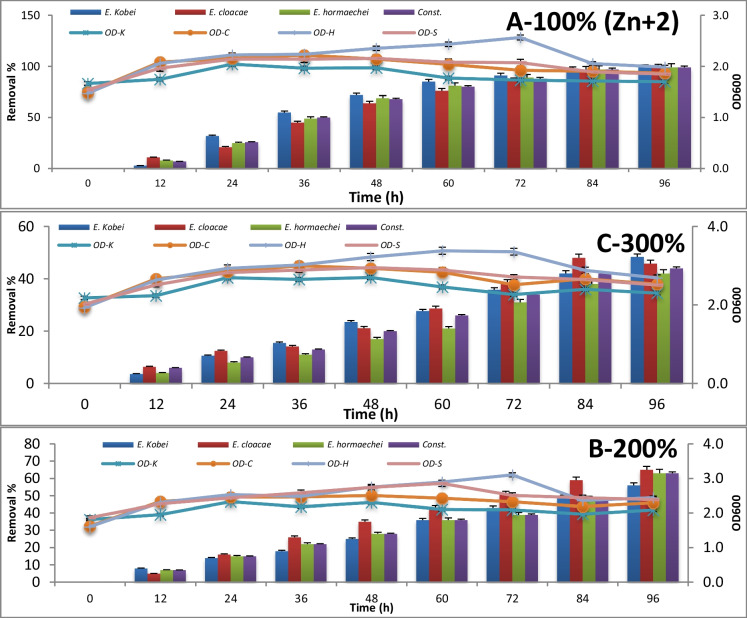
Consortium removal of Zn^+2^ using *Enterobacter* isolates at different concentrations

The genetic identification of bacterial isolates was explained using Biosystem 3130 genetic analyzers; this analyzer produced 16S rRNA bases by 1414, 1399, and 1407 for S4, S5, and S7 isolates. The gene bases were identified to genus level (up to 99% identity or better), using available GenBank databases. According to 16S rRNA gene sequence analysis of isolate S4, S5, and S7 compared to Blast which provided the highest homology. The results showed that the isolates under study were similar to *Enterobacter* spp. and recorded in the NCBI database as *E. kobei* OM144907 (S4), *E. cloacae* OM180597 (S5), and *E. hormaechei* OM181067 (S7) with 99.58, 99.79, and 99.86% similarity percentage. All identified strains are deposited in WDCM with reference numbers SCUF0000311, SCUF0000312, and SCUF0000313 for *E. kobei* OM144907, *E. cloacae* OM180597, and *E. hormaechei* OM181067, respectively. As shown in supplementary file b figure 3S-b-i, the identified strain (*E. kobei* SCUF0000311) and *E. kobei* (NZ-JZYH01000051) were in the same clade by 0.72 points with 99.58 % similarity. The most similar strains to our identified strain were *Pantoea agglomerans* (MW876168), *Enterobacter* sp. (KU986680), *E. ludwigii* (MN636653), *E. kobei* (NZ-LEEC01000015), and *P. agglomerans* (MW876157). Also, *E. cloacae* (SCUF0000312) phylogeny is designed in supplementary file b figure 3S-b-ii by similarity 99.79%. Our identified isolates were most similar to *Bacterium* sp. (MK823507), *E. cloacae* (KU297784), *E. ludwigii* (MH001397), *Enterobacter* sp. (MN540103), *P. agglomerans* (FJ592995), and *Enterobacter* sp. (GQ169799). About *E. hormaechei* (SCUF0000313), it attained 99.86% similarity with *Enterobacter* sp. (MF401327). *E. hormaechei* (MW582664), *E. hormaechei* (MW435507), *E. hormaechei* (MW582678), *Bacterium* sp. (MZ045739), *E. hormaechei* (MT941037), and *E. hormaechei* (MN428803) which were closely similar to our identified strain (supplementary file b figure 3-b-iii).

### Minimum inhibition concentration of tolerant samples

The MICs of the seven metal ions against the studied bacterial isolates were shown in Figure 2. The growth rate of the bacteria exhibited a gradual increase by decreasing metal concentration relative to the control. The concentration of Zn^+2^, Fe^+2^, Pb^+2^, Co^+2^, Mn^+2^, Ni^+2^, and Cd^+2^ were 0.1, 1, 10, 15, 25, and 35 mmol/l. The MIC for *E. kobei* and *E. cloacae* against metals ion were demonstrated by 25 mmol/l for Ni^+2^, 15 mmol/l for Fe^+2^ and Mn^+2^ and 10mmol/l for Zn^+2^, Pb^+2^, Co^+2^, and Cd^+2^. On the other hand, the MIC for *E. hormaechei* against metals ion was demonstrated by 15 mmol/l for Ni^+2^, Fe^+2^, and Mn^+2^ and 10 mmol/l for Zn^+2^, Pb^+2^, Co^+2^, and Cd^+2^. The growth pattern appears to suggest tolerance development or adaptation of bacteria to the presence of heavy metals.

### Sediment sieve analysis with carbonate and organic matter

The bioremediation capacity depends on the geochemistry of the drain pathway, which by analysis is described as very coarse silty medium sand with a muddy texture. The percentage of gravel, sand, and mud was 0.00%, 84.4%, and 15.6%, respectively. Grain size statistical parameters such as mean size (Mz), kurtosis (KG), and skewness (SKI) are 2.622, 0.799, and 0.165. The total organic matter % and total carbonate % of the drain were 31.5 and 20.54 % (Table 3). On the other hand, the analysis of marine sediment achieved poorly sorted very coarse sand by gravel, sand, and mud 0.00, 98.6, and 1.4 with mean size (Mz), kurtosis (KG), and skewness (SKI) 1.099, 0.21 and 0.71, respectively.

**Table 3:** Results of the grain size analysis, the estimated geologic constituents, total organic matter %, and total carbonate %

Stations	Grain size analysis									
	Gravel%	Sand%	Mud%	MZ	SK	KG	TG	SN	SD	S
*Adabiya drain*	0.00	84.4	15.6	2.622	0.165	0.799	Muddy sand	Very coarse silty medium sand	Fine	Poorly sorted
*Marine water*	0.00	98.6	1.4	1.099	0.21	0.71	Sand	Poorly sorted very coarse sand	coarse	Poorly sorted
Stations	Total organic matter % & total carbonate%									
	Total organic matter %					Total carbonate %				
*Adabiya drain*	31.5					20.54				
*Marine water*	2.99					79.93				
Stations	Leachable heavy metals (μg/g) in sediment									
	Cu		Zn	Fe	Pb	Co	Mn	Ni	Cd	
*Adabiya drain*	12.49		1.68	2.71	92.06	12.48	5.84	72.06	3.80	
*Marine water*	5.6		5.3	29.5	5.9	1.64	24.1	2.63	1.6	

*Mz* mean size, *SK* skewness, *KG* kurtosis, *TG* textural group, *SN* sediment name, *SD* sediment description, *S* sorting

The metal concentrations of drain and marine sediment are different considering the sources of the pollutants. The concentration of Fe^+2^, Mn^+2^, Zn^+2^, Pb^+2^, Cd^+2^, Ni^+2^, and Co^+2^ metals were 2.71, 5.84, 1.68, 92.06, 3.80, 72.06, and 12.48 mg/g; nevertheless, in marine sediment, the Fe^+2^, Mn^+2^, Zn^+2^, Pb^+2^, Cd^+2^, Ni^+2^, and Co^+2^ concentrations were 5.6, 5.3, 29.5, 5.9, 1.64, 24.1, 2.63, and 1.6 μg/g, respectively (Table 3).

### Application of bacterial strains for bioremediation of drain sewage water

**Fig. 4:**
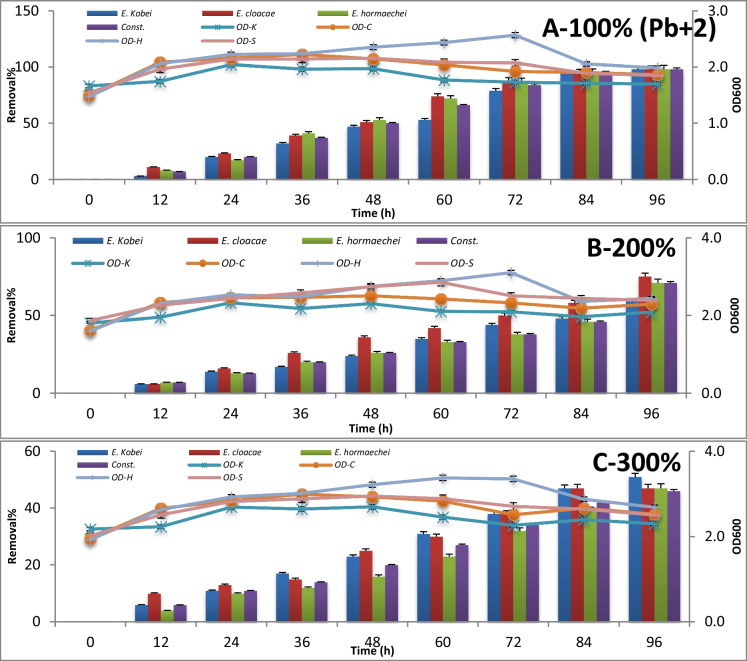
Removal percentage of Pb^+2^ at 100 %, 200 %, and 300 % concentrations after 96 h of incubation

**Fig. 5:**
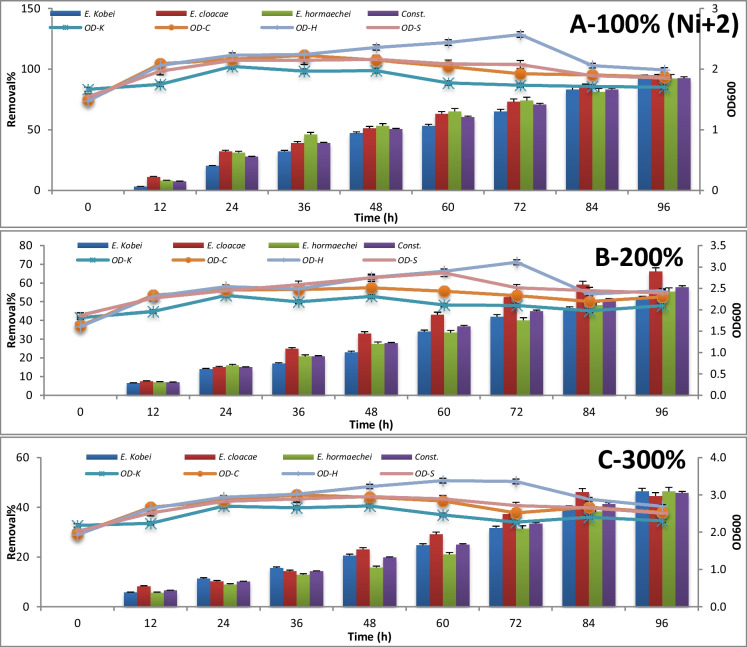
Nickel removal percentage using *Enterobacter* strains after 96 h of incubation at different concentrations

**Fig. 6:**
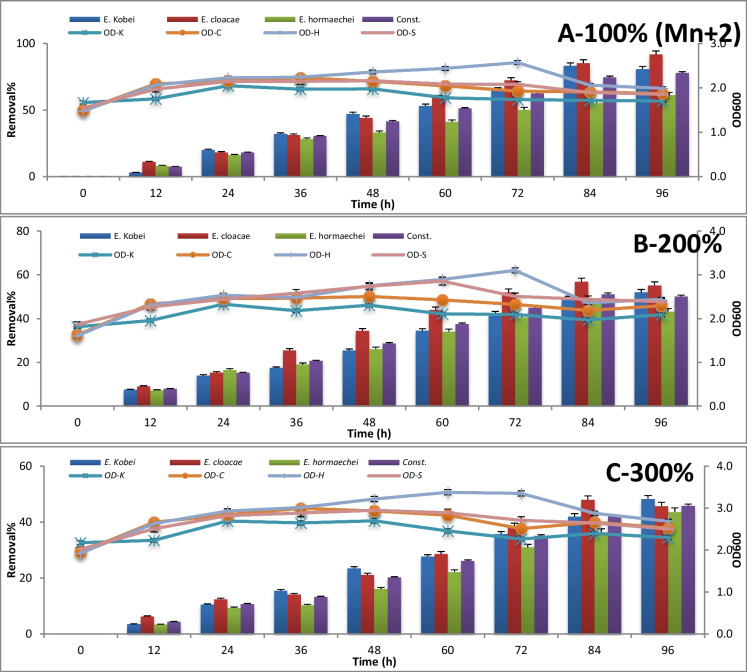
The consortium test for Mn^+2^ removals using *Enterobacter* strains

**Fig. 7:**
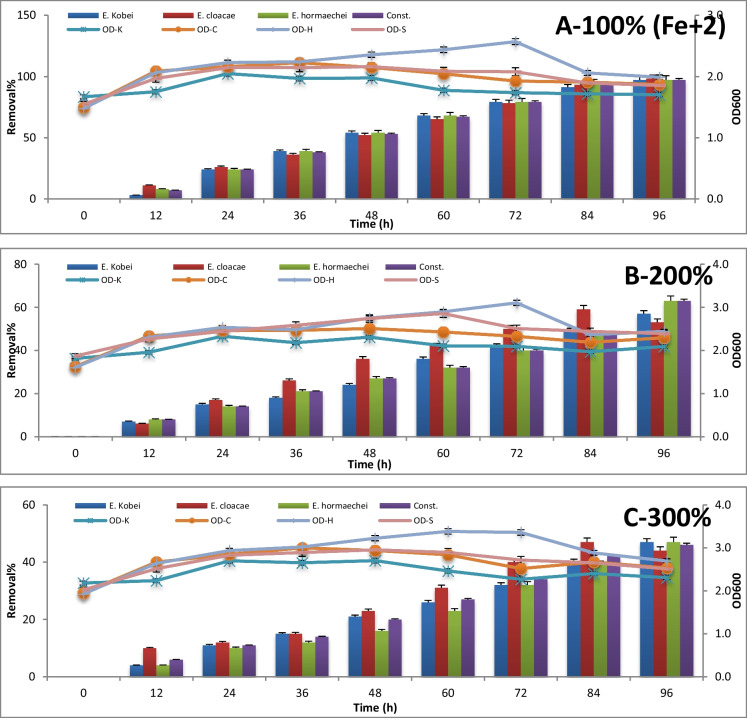
The removal % and optical density of different *Enterobacter* strains for Fe^+2^ bioremediation

**Fig. 8:**
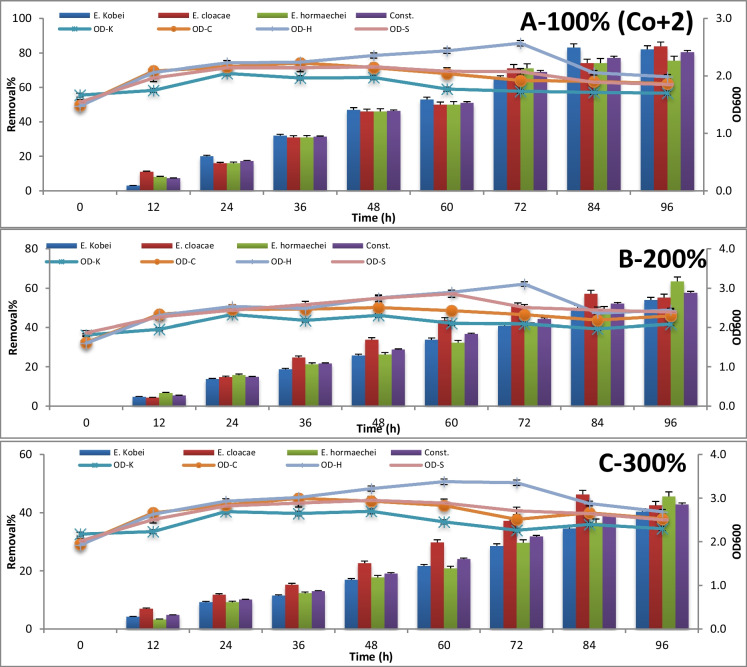
The bioremediation of cobalt at 100%, 200%, and 300% concentrations after 96h

**Fig. 9:**
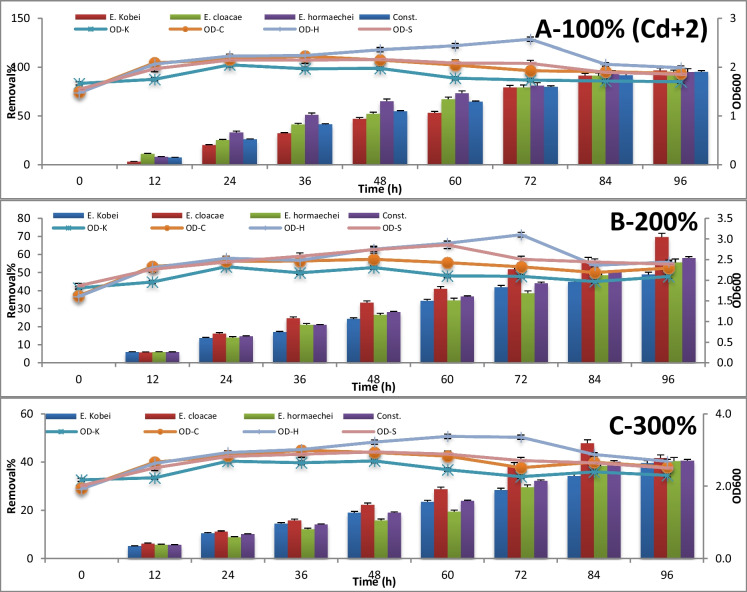
Cadmium removal % after 96 h of incubation using *Enterobacter* strains

The consortium test for metal removal at 100%, 200%, and 300% concentrations is expressed by absorbance measuring at 600 nm and metal concentration measuring using atomic absorption every 12 h until 96 h of incubation. The results are represented in Figures 3, 4, 5, 6, 7 , 8 and 9 for Zn^+2^, Pb^+2^, Ni^+2^, Mn^+2^, Fe^+2^, Co^+2^, and Cd^+2^, respectively.

As illustrated in Figure 3, starting with no remediation, where the concentration before any treatment was 54.2 μg/l, approximately a complete removal of Zn^+2^ with a removal percentage of 99% was achieved after 96-h incubation period for the examined strains and their consortium as well. However, *E. cloacae* removed about 65% of the doubling load of Zn^+2^ in the polluted sample after an incubation period of 96 h. In addition, it removed about 47% of Zn^+2^ from the tripling concentration of the sample after 84-h incubation time within the stationary growth phase in all cases.

Having an initial concentration of 15.12 μg/l of Pb^+2^, all tested potential strains exhibited a high efficiency of Pb^+2^ removal (99%) after 96-h incubation time during a stationary growth phase, even with their consortium. But, *E. cloacae* was the one that succeeded in removing about 75% of Pb^+2^ from doubling the concentration of Pb^+2^ after 96-h incubation period, while *E. kobei* was the one that removed about 51% of the tripling load of Pb^+2^ concentration in the polluted sample after 96 h within the stationary growth phase (Figure 4).

In Figure 5, about 93% removal of Ni^+2^ from the initial concentration load 3.32 μg/l was reported after incubation of the polluted sample with the potential strains and their consortium as well for 96-h incubation period. Yet, *E. cloacae* alone showed a great potential to remove Ni^+2^ from doubling and tripling concentration of Ni^+2^ by 66% and 46% after 96 and 84 h, respectively, within its stationary growth phase.

In Figure 6, starting with a concentration of 0.484 μg/l of Mn^+2^ as the initial loading sample, *E. cloacae* alone showed a remarkable efficiency to remove Mn^+2^ (91%) after 96-h incubation time during the stationary growth phase, and such efficiency was kept steady with doubling and tripling load of Mn^+2^ concentration in the sample with removal percentage 57% and 48%, respectively, after 84-h incubation time during the stationary growth phase.

Polluted sample with Fe^+2^ having a concentration of 44.76 μg/l was bio-remediated to approximately 97% removal with equal efficiency for all tested strains and their consortium after 96-h incubation time with the stationary growth phase of them. Nevertheless, 63% removal of doubling the Fe^+2^ concentration after 96-h incubation time was reported by *E. hormaechei*. Yet, *E. cloacae* were the supreme of removing Fe^+2^ in all incubation periods except 96-h incubation measurement. On the other side, equal removal efficiency (47%) of tripling the Fe^+2^ concentration from the polluted sample was done by the three tested potential strains in addition to their consortium during the stationary phase of their growth (Figure 7).

In Figure 8, the highest removal of Co^+2^ (84%), where the initial loading concentration (100%) was 0.765 μg/l, was achieved by *E. cloacae* after 96-h incubation period; however, *E. kobei* removed about 83%, as well, of polluted sample from Co^+2^ after 84 h, doubling the concentration of Co^+2^; *E. hormaechei* succeeded to remove about 63% of Co^+2^ after 96 h, while *E. cloacae* removed about 57% after 84 h. with increasing the concentration of pollutant representing as Co^+2^ to triple the concentration in the original polluted sample; *E. cloacae* removed about 46% of Co^+2^ after 48 h. All successive removal was determined during the stationary phase of all tested microbial growth.

In Figure 9, the highest removal percentage of Cd^+2^ starting from loading concentration 1.142 μg/l was achieved between 94 and 96%) from a polluted sample using the three potential strains separately and their consortium as well after 96-h incubation period, where the stationary phase of their growth has occurred. However, with doubling the Cd^+2^ concentration in the polluted sample, *E. cloacae* expressed the highest efficiency of removal percentage (70%) after 96-h incubation time within the stationary growth phase. With more loading of Cd^+2^ concentration in the treated sample reaching tripling the original concentration, *E. cloacae* removed about 48% of Cd^+2^ after 48-h incubation time within the stationary growth phase. The data of solo and consortium species removal is illustrated in the supplementary file table 1S-a, 2S-a, 3S-a, and 4S-a.

The microbial bioremediation of Zn^+2^, Pb^+2^, Ni^+2^, Mn^+2^, Fe^+2^, Co^+2^, and Cd^+2^ using (i) *E. kobei* Wdcm scuf0000311, (ii) *E. cloacae* Wdcm scuf0000312, (iii) *E. hormaechei* Wdcm scuf0000312, and (iv) consortium culture of previously applied strains on different concentrations (a) polluted sample representing 100%; (b) doubled heavy metal concentration in the same sample representing 200%, of the original polluted sample; and (c) tripled concentration of heavy metal concentration representing 300%, of the original polluted sample. The standard deviation was calculated for each record as SD 0.05.

## Discussion

The more industrial activities discharged without any treatment, the more pollution and toxic effects on the relevant surrounding environment get. This would be the major reason for spreading the pollution. Time-consuming and charging a high cost to mechanically remove the heavy metal contaminants result in the deviation of scientists’ thoughts towards a practical solution that focuses on using bacterial cells possessing multiple mechanisms for heavy metal removal. The current study succeeded in isolating and purifying three bacterial isolates genetically identified as *E. kobei* (SCUF0000311), *E. cloacae* (SCUF0000312), and *E. hormaechei* (SCUF0000313) and having a potential resistance to high concentrations of Zn2+, Fe2+, Pb2+, Co2+, Mn2+, Ni2+, and Cd2+ metals using accumulation property. Lately, Banerjee et al. (2015) have reported *E. cloacae* as a potent strain to accumulate lead, cadmium, and nickel, whereas Bestawy et al. (2013) have removed different heavy metals from contaminated domestic–industrial effluent with aid of eight resistant indigenous bacteria isolated from activated sludge as well as Rahman et al. (2015) who have reported the possibility of chromium removal from local human activities (industries, agriculture, forest farming, mining, and metallurgy) using *E. cloacae* B2-D HA. These manuscripts has been studied and identified to have various resistance mechanisms including transport channels and compartmentalization within the cell (Banerjee et al., 2015; Bestawy et al., 2013; Rahman et al., 2015).

Varied heavy metal removal mechanisms have been reported such as bacterial cell wall attachment, siderophores production for chelation, and heavy metal metabolic transportation (Ahemad, 2012; Schalk et al., 2011). As reported in the current study, the minimum inhibitory concentration of *E. kobei* (SCUF0000311), *E. cloacae* (SCUF0000312), and *E. hormaechei* (SCUF0000313) against Ni2+, Fe2+, and Mn2+ was recorded to be 15mmol/l compared to Zn2+, Pb2+, Co2+, and Cd2+ with 10mmol/l. Previous studies have reported MIC of *Bacillus carotarum*, *B. cereus*, *B. lentus*, and *B. licheniformis* isolated from Jabalpur, India, against lead, zinc, and chromium by 1% and 0.01% (Gupta Mahendra et al., 2014). Moreover, *E. cloacae* B2-DHA has recorded MIC value against chromium as 1000 μg/mL^−1^ (Rahman et al., 2015).

Our study encompasses a vast amount of information about the bioremediation process of a considerable number of heavy metals. This study approached the measurement of bioremediation in an innovative way by experimenting with the removal of heavy elements separately by *E. kobei* (SCUF0000311) and *E. cloacae* (SCUF0000312) and *E. hormaechei* (SCUF0000313) and by combining the three strains into one sample and testing them individually. This method had not been previously addressed by any of the previous scientists, as we have in our current study, resulting in a precise analysis of heavy element removal percentages using the mentioned strains. Poornima et al. (2014) and Pandey et al. (2011) achieved a similar concept in our study without our sequence work by isolating *E. coli* PS01 and *Bacillus* sp., both of which can withstand high concentrations of chromium, lead, and arsenic (Pandey et al., 2011; Poornima et al., 2014). In the study conducted by Rani et al. (2010), three bacterial isolates, namely, *Bacillus* sp., *Pseudomonas* sp., and *Micrococcus* sp., were isolated, and their bioaccumulation capacities were reported as follows: 69.34% for copper, 90.41% for cadmium, and 84.27% for lead. Similarly, Ahemad and Malik (2011) documented the accumulation of various metals such as lead, chromium, mercury, and zinc by multiple bacterial species isolated from agricultural fields and wastewater. In contrast, our study revealed that the bacterial strain *E. cloacae* B1 exhibited significantly higher lead accumulation capacity compared to cadmium and nickel.

As previously documented by numerous researchers, various bacterial strains have been shown to possess metal-reducing capabilities, demonstrating their potential for biotransformation and the ability to reduce varying amounts of chromium in the medium. Thacker et al. (2007) reported the existence of a Gram-negative strain of *Brucella* sp. with the capacity to reduce chromium levels in contaminated sources. This strain’s resistance to high concentrations of metals and its proficiency in reducing this toxic metal make it a promising candidate for bioremediation purposes. Additionally, scientists can identified and characterized three highly efficient metal-reducing bacterial strains, namely *Bacillus cereus*, *Bacillus fusiformis*, and *Bacillus sphaericus*, which were isolated from metal-polluted landfills and evaluated for in vitro metal reduction (Desai et al., 2008; Zhang & Wang, 2021). This aligns with what we have reached through our current study, which allows us to assert the potential use of microbes for the removal of heavy elements from industrial wastewater.

Metal concentrations of Fe^+2^, Mn^+2^, Zn^+2^, Pb^+2^, Cd^+2^, Ni^+2^, and Co^+2^ were 2.71, 5.84, 1.68, 92.06, 3.80, 72.06, and 12.48 μg/g in a sediment layer, respectively. Maslennikova et al. (2012) have indicated that within the smaller grain size where the higher surface area exists, the more heavy metal content to be there. Also, previous studies have revealed that organic matter hydrolysis in bottom sediments could be another source for adsorbing heavy metals on sediment grains that would be later liberated into the surrounding environment via desorption, microbial activities, substitution, or dissolution due to any alter of pH levels or redox potential processes, which in turn would reflect on water quality and surrounding aquatic ecosystem (Maslennikova et al., 2012; Yang et al., 2020; Zamani Hargalani et al., 2014).

The nature of the drain sediment was different from marine, which explains the accumulation of pollutants in the drain sediment leading to the appearance of soil as clay and muddy allowing for heavy metal accumulation. Dixit et al. (2015) reported that a heavily polluted soil allows water droplets to adhesion to the hydrophobic layer, and this prevents the wetting of the soil aggregates (Dixit et al., 2015).

During this study, the bacterial strains that were isolated in this study area could not reduce the field metal percentage. By the availability of suitable conditions for bacterial growth, isolated strains were adapted for metal high percentages in the presence of growth factors and nutrition. It is noteworthy that the nature of the clay soil in the drain area does not allow aerobic bacterial growth but allows anaerobic bacteria enumeration (Chen et al., 2021). Wellsbury et al. (2002) recognized that small pores restrict bacteria movement and activity, limit nutrient transport, diminish space availability, slow the rate of division, and lead to reduced biodiversity. So, the most species of bacteria isolated in this study were *Enterobacter* sp. (Chen et al., 2021; Wellsbury et al., 2002).

It was observed that toxic sediments including decaying organic matters play a vital role in controlling the binding of existing heavy metals to sediment grains as well as the bioavailability of heavy metals with different toxicity and safety levels. However, quantitative measurement of organic matter content is rarely analyzed in contaminant studies. On the other side, it was found that the composition of organic matter varies widely within the available organic matter content offering diverse effects (Baran & Tarnawski, 2015; Chiriluș et al., 2022)**.**

The concept of microbial heavy metal bioremediation has been evaluated via biosorption, bioaccumulation, bioprecipitation, or biomineralization. Those are the milestones of any microbial remediation so far, and the metabolic pathway of each differs from microbial strain to another (Lin & Lin, 2005; Sreedevi et al., 2022). The current study has revealed that, upon studied strains, *Enterobacter* spp. include potent strains for heavy metal bioremediation. Out of three examined *Enterobacter* strains (*E. kobei* SCUF0000311, *E. cloacae* SCUF0000312, and *E. hormaechei* SCUF0000313), *E. cloacae* (SCUF0000312) proved to be the one with high capability to bioremediate a broad spectrum of heavy metals including the current study with the privilege to bioremediate high concentrations as doubling and tripling the original waste concentration with efficient time factor in comparison with other previous studies of *Enterobacter* spp. This study showed that MIC for *E. kobei* and *E. cloacae* against (Ni^+2^), (Mn^+2^, Fe^+2^) and (Zn^+2^, Pb^+2^, Co^+2^, Cd^+2^) were 25, 15, and 10 mmol/l, respectively, while MIC for *E. hormaechei* against (Mn^+2^, Ni^+2^, Fe^+2^) and (Zn^+2^, Pb^+2^, Co^+2^, Cd^+2^) were 15 and 10 mmol/l.

*Enterobacter* species have been registered by Fadzli et al. (2021) as a potent species for heavy metal remediation recording high removal efficiency of Pb^+2^, Cd^+2^, and Cr^+3^ as 90.14, 88.00, and 90.34%, respectively, within 30-day incubation (Fadzli et al., 2021).

*E. cloacae* have been observed as an efficient microbial biosorbent giving a high uptake concentration of Pb^+2^ (2.3 mmoles) from the initial concentration (7.2 mmol) (Kang et al., 2015). In addition, *E. cloacae* have been found to have MIC (1000 ug/ml) with Cr^+2^ having a mechanism of intracellular accumulation of heavy metal and recording 81% of Cr^+2^ reduction from the liquid medium after 120-h incubation period (Rahman et al., 2015). Banerjee et al. (2015) have reported that the MIC of *E. cloacae* towards Pb^+2^, Cd^+2^, and Ni^+2^ was 1100, 900, and 700 ppm, respectively. Consequently, the high efficiency of bioaccumulation in percentage with those heavy metals has been recorded as Pb^+2^ (95.25%), Cd^+2^ (64.17%), and Ni^+2^ (36.77%) (Banerjee et al., 2015). Moreover, Abdollahi et al. (2020) have reported that *E. cloacae* had MIC 3000 ug/ml and 50 ug/ml against Pb^+2^ and Cd^+2^ with accumulation capacity 45ug Pb^+2^/ml and 30ug Cd^+2^/ml. Also, Ghosh et al. (2022) have reported that *E. cloacae* expressed a high potency of tolerance towards high concentrations of Cd^+2^ (4000 μg/ml), Pb^+2^ (3312 μg/ml), and As^+3^(1500 μg/ml), where the removal efficiency of Cd^+2^ was recorded 72.11% (Abdollahi et al., 2020; Ghosh et al., 2022).

With a few reports on the capability of *E. hormaechei* (SCUF0000313) and *E. kobei* (SCUF0000311) to bioremediate heavy metals, Heidari et al. (2020) have found that *E. hormaechei* exposed a high efficiency of uptake towards Ni^+2^ than Pb^+2^ and Cd^+2^. Abdollahi et al. (2020) found that *E. kobei* had MIC 3000 μg/ml and 50 μg/ml towards Pb^+2^ and Cd^+2^, respectively, in addition to an accumulation capacity of 25 μg Pb^+2^/ml and 20μg Cd^+2^/ml (Abdollahi et al., 2020; Heidari et al., 2020).

Overall, among the tested potential *Enterobacter* spp. for heavy metal remediation, *E. cloacae* (SCUF0000312) has proved to be the most potent strain for water treatment in a sufficient way.

## Conclusion

In conclusion, the study presented here highlights the critical role that bacterial strains, particularly *Enterobacter* spp., can play in the bioremediation of heavy metals from polluted environments. The traditional methods for removing heavy metal contaminants are often time-consuming and costly. The research conducted in this study isolated and identified three *Enterobacter* strains, namely, *E. kobei* (SCUF0000311), *E. cloacae* (SCUF0000312), and *E. hormaechei* (SCUF0000313), which exhibited high resistance to a range of heavy metals, including zinc, lead, cobalt, cadmium, and others. Of these strains, *E. cloacae* (SCUF0000312) emerged as particularly effective in bioremediation efforts, surpassing other *Enterobacter* species in terms of both efficiency and capacity. Different heavy metal removal mechanisms have been reported, including bacterial cell wall attachment, siderophores production for chelation, and heavy metal metabolic transportation. Furthermore, this study introduced an innovative approach to assessing heavy metal removal by experimenting with individual strains and their combined effectiveness. This method allowed for a precise analysis of heavy metal removal percentages using these specific bacterial strains, which had not been previously explored in such detail. The study area is characterized by its clay and muddy composition, which presented challenges for aerobic bacterial growth. However, anaerobic bacterial enumeration was possible, underscoring the importance of environmental factors in shaping bacterial activity and metal removal capabilities. The findings from this study contribute to the growing body of research on microbial bioremediation and emphasize the potential of *Enterobacter* spp., particularly *E. cloacae* (SCUF0000312), as valuable tools in addressing heavy metal pollution in industrial wastewater. The versatility and efficiency demonstrated by these bacterial strains offer promising avenues for the development of sustainable and cost-effective solutions to mitigate the harmful effects of heavy metal contamination on the environment. Continued research in this field can lead to more effective bioremediation strategies that help protect ecosystems and human health.

### Supplementary information

ESM 1 (DOCX 40 kb)

ESM 2 (DOCX 924 kb)

## Data Availability

The raw data supporting the conclusions of this manuscript would be available by the authors, without undue reservation, to any qualified researcher.

## Abbreviations

Zn^2+^: zinc
Fe^2+^: iron
Pb^2+^: lead
Co^2+^: cobalt
Mn^2+^: manganese
Ni^2+^: nickel
Cd^2+^: cadmium
DNA: deoxyribonucleic acid
*E. kobei*: *Enterobacter kobei*
*E. cloacae*: *Enterobacter cloacae*
*E. Hormaechei*: *Enterobacter hormaechei*
K_2_HPO_4_: dipotassium hydrogen orthophosphate
KH_2_PO_4_: potassium dihydrogen orthophosphate
NaCl: sodium chloride
NH_4_NO_3_: ammonium nitrate
MgSO_4_: magnesium sulfate
Pb(CH_3_COO)_4_: lead tetraacetate
CuSO_4_: copper sulfate
ZnSO_4_: zinc sulfate
Co(NO_3_)_2_·6 H_2_O: cobalt nitrate anhydrase
CaCl_2_: calcium chloride
H_2_O: water
h: hours
°C: degree Celsius
rpm: rotation per minute
OD: optical density
CFU/mL: colony-forming unit per mill
μl: micron
mM: mill mole
MIC: minimum inhibition concentration
ONBG: O-nitrophenyl-beta-D-galactopyranoside
rDNA: ribosomal deoxyribonucleic acid
APDC: ammonium pyrrolidine dithiocarbamate
MIBK: methyl isobutyl ketone
HNO_3_: nitric acid
HClO_4_: perchloric acid
FAAS: flame atomic absorption spectroscopy
Ø: one phi
MZ: mean size
δ: sorting
SKI: skewness
KG: kurtosis
CO3: carbonate
TOM: total organic matter
