# Supplementary material for: Bioremediation of industrial wastewater heavy metals using solo and consortium Enterobacter spp
Source: Environ Monit Assess. 2023 Oct 23;195(11):1357. doi: 10.1007/s10661-023-11951-x (PMC10593623; doi:10.1007/s10661-023-11951-x)
Supplement: Supplementary file 1 — (DOCX 40 kb) [file 10661_2023_11951_MOESM1_ESM.docx]

|  | **List of tables** |
| --- | --- |
| **Table 1S-a** | Application of three bacterial isolates against 100% of drainage concentration |
| **Table 2S-a** | Bacterial consortium and solo application against 200% of drainage concentration |
| **Table 3S-a** | Solo and consortium of bacterial strains against 300% of drainage concentration |
| **Table 4S-a** | Consortium and solo application of bacteria against 100%, 200% and 300% of drainage concentration |

**Table 1s-a:** Application of three bacterial isolates against 100% of drainage concentration

|  | Removal % of *E. Kobei* Wdcm scuf0000311 isolate by using 100% drainage concentration | | | | | | | | |
| --- | --- | --- | --- | --- | --- | --- | --- | --- | --- |
|  | **Befor R** | **12 h** | **24h** | **36 h** | **48 h** | **60 h** | **72 h** | **84 h** | **96 h** |
| **Zn** | 0 | 3 | 32 | 55 | 72 | 85 | 91 | 97 | 98.58 |
| **Fe** | 0 | 3 | 24 | 39 | 54 | 68 | 79 | 91 | 97.40 |
| **Pb** | 0 | 3 | 20 | 32 | 47 | 53 | 79 | 94 | 97.65 |
| **Co** | 0 | 3 | 20 | 32 | 47 | 53 | 65 | 83 | 81.96 |
| **Mn** | 0 | 3 | 20 | 32 | 47 | 53 | 65 | 83 | 80.58 |
| **Ni** | 0 | 3 | 20 | 32 | 47 | 53 | 65 | 83 | 92.61 |
| **Cd** | 0 | 3 | 20 | 32 | 47 | 53 | 79 | 91 | 96.41 |
|  | Removal % of *E. cloacae* Wdcm scuf0000312 isolate by using 100% drainage concentration | | | | | | | | |
|  | **Befor R** | **12 h** | **24h** | **36 h** | **48 h** | **60 h** | **72 h** | **84 h** | **96 h** |
| **Zn** | 0 | 11 | 21 | 45 | 64 | 76 | 86 | 97 | 99.05 |
| **Fe** | 0 | 11 | 26 | 36 | 52 | 65 | 78 | 93 | 97.69 |
| **Pb** | 0 | 11 | 23 | 39 | 51 | 74 | 88 | 95.3 | 98.07 |
| **Co** | 0 | 11 | 16 | 31 | 46 | 50 | 71 | 74 | 83.66 |
| **Mn** | 0 | 11 | 18 | 31 | 44 | 59 | 72 | 85 | 91.32 |
| **Ni** | 0 | 11 | 32 | 39 | 51 | 63 | 73 | 85 | 92.58 |
|  | Removal % of *E. hormaechei* Wdcm scuf0000313 isolate by using 100% drainage concentration | | | | | | | | |
| **Zn** | **Befor R** | **12 h** | **24h** | **36 h** | **48 h** | **60 h** | **72 h** | **84 h** | **96 h** |
|  | 0 | 8 | 25 | 49 | 69 | 81 | 89 | 97.5 | 98.66 |
| **Fe** | 0 | 8 | 24 | 39 | 54 | 68 | 79 | 94 | 97.22 |
| **Pb** | 0 | 8 | 17 | 41 | 53 | 72 | 87 | 95 | 98.33 |
| **Co** | 0 | 8 | 16 | 31 | 46 | 50 | 71 | 74 | 75.29 |
| **Mn** | 0 | 8 | 16 | 28 | 33 | 41 | 50 | 55 | 60.95 |
| **Ni** | 0 | 8 | 31 | 46 | 53 | 65 | 74 | 81 | 92.07 |
| **Cd** | 0 | 8 | 33 | 51 | 65 | 73 | 81 | 93 | 94.83 |
| **Cd** | 0 | 11 | 25 | 41 | 52 | 67 | 79 | 91 | 93.96 |

**Table 2:** Bacterial consortium and solo application against 200% of drainage concentration

|  | Removal % of *E. Kobei* Wdcm scuf0000311 isolate by using 200% drainage concentration | | | | | | | | |
| --- | --- | --- | --- | --- | --- | --- | --- | --- | --- |
|  | **Befor R** | **12 h** | **24h** | **36 h** | **48 h** | **60 h** | **72 h** | **84 h** | **96 h** |
| **Zn** | 0 | 7.5 | 13.5 | 18 | 25 | 35.5 | 43 | 49 | 56 |
| **Fe** | 0 | 7 | 14.5 | 17.5 | 24 | 36 | 42 | 48.5 | 57 |
| **Pb** | 0 | 6 | 14 | 17 | 23.5 | 35 | 44 | 48 | 59 |
| **Co** | 0 | 4.819 | 13.84 | 18.85 | 25.86 | 33.87 | 40.89 | 49.90 | 53.91 |
| **Mn** | 0 | 7.49 | 14 | 17.50 | 25.50 | 34.50 | 42.19 | 48.99 | 51.99 |
| **Ni** | 0 | 6.48 | 13.99 | 16.99 | 22.99 | 33.99 | 41.99 | 45.99 | 51.49 |
| **Cd** | 0 | 5.98 | 13.76 | 16.97 | 24.28 | 34.31 | 41.83 | 44.84 | 48.85 |
|  | Removal % of *E. cloacae* Wdcm scuf0000312 isolate by using 200% drainage concentration | | | | | | | | |
|  | **Befor R** | **12 h** | **24h** | **36 h** | **48 h** | **60 h** | **72 h** | **84 h** | **96 h** |
| **Zn** | 0 | 5.2 | 16.5 | 26 | 35 | 43 | 51 | 59 | 65.06 |
| **Fe** | 0 | 5.8 | 17.3 | 25.5 | 36 | 42 | 50 | 58.5 | 52.53 |
| **Pb** | 0 | 5.6 | 16.2 | 25.8 | 36.4 | 42.4 | 49.6 | 58 | 75.17 |
| **Co** | 0 | 4.42 | 14.83 | 24.85 | 33.87 | 43.69 | 50.91 | 57.12 | 55.24 |
| **Mn** | 0 | 8.99 | 15.29 | 25.49 | 34.39 | 43.99 | 51.99 | 56.79 | 55.14 |
| **Ni** | 0 | 7.48 | 14.98 | 24.79 | 32.99 | 42.99 | 52.39 | 58.99 | 66.03 |
| **Cd** | 0 | 5.80 | 16.19 | 24.69 | 33.29 | 40.99 | 51.79 | 56.69 | 69.64 |
|  | Removal % of *E. hormaechei* Wdcm scuf0000313 isolate by using 200% drainage concentration | | | | | | | | |
|  | **Befor R** | **12 h** | **24h** | **36 h** | **48 h** | **60 h** | **72 h** | **84 h** | **96 h** |
| **Zn** | 0 | 7 | 15 | 22 | 28 | 36 | 39 | 48 | 62.52 |
| **Fe** | 0 | 8 | 14 | 21 | 26.5 | 32 | 40 | 47 | 63.18 |
| **Pb** | 0 | 7.3 | 13 | 20 | 26 | 33 | 38 | 46 | 71.32 |
| **Co** | 0 | 6.82 | 15.84 | 21.35 | 26.36 | 32.37 | 40.88 | 48.90 | 63.42 |
| **Mn** | 0 | 7.19 | 16.49 | 18.99 | 25.99 | 33.99 | 40.49 | 47.49 | 43.13 |
| **Ni** | 0 | 6.98 | 15.98 | 20.78 | 27.49 | 33.49 | 39.99 | 47.99 | 55.30 |
| **Cd** | 0 | 5.99 | 13.99 | 20.99 | 26.49 | 34.49 | 38.49 | 48.49 | 55.50 |

**Table 3:** Solo and consortium of bacterial strains against 300% of drainage concentration

|  | Removal % of *E. Kobei* Wdcm scuf0000311 isolate by using 300% drainage concentration | | | | | | | | |
| --- | --- | --- | --- | --- | --- | --- | --- | --- | --- |
| **Zn** | **Befor R** | **12 h** | **24h** | **36 h** | **48 h** | **60 h** | **72 h** | **84 h** | **96 h** |
|  | 0 | 6.6 | 10.1 | 13.7 | 20.6 | 25.5 | 32 | 41 | 43.6 |
| **Fe** | 0 | 4.4 | 10.6 | 15 | 20.5 | 26.4 | 32.2 | 40 | 46.65 |
| **Pb** | 0 | 5.6 | 10.8 | 16.8 | 22.5 | 30.6 | 38.3 | 46.6 | 50.65 |
| **Co** | 0 | 4.38 | 9.35 | 11.6 | 17 | 21.7 | 28.6 | 34.6 | 40.43 |
| **Mn** | 0 | 3.6 | 10.6 | 15.5 | 23.5 | 27.7 | 35.8 | 42.04 | 48.36 |
| **Ni** | 0 | 5.8 | 11.4 | 15.7 | 20.6 | 24.7 | 31.6 | 39.6 | 46.42 |
| **Cd** | 0 | 5.3 | 10.7 | 14.6 | 19.1 | 23.6 | 28.5 | 34.3 | 39.65 |
|  | Removal % of *E. cloacae* Wdcm scuf0000312 isolate by using 300% drainage concentration | | | | | | | | |
|  | **Befor R** | **12 h** | **24h** | **36 h** | **48 h** | **60 h** | **72 h** | **84 h** | **96 h** |
| **Zn** | 0 | 7.49 | 10.26 | 13.55 | 23.11 | 30.53 | 38.46 | 47.66 | 44.6 |
| **Fe** | 0 | 9.54 | 11.98 | 14.96 | 22.77 | 31.13 | 39.46 | 46.55 | 44.3 |
| **Pb** | 0 | 10.29 | 12.95 | 14.85 | 24.64 | 30.36 | 38.32 | 46.76 | 46.9 |
| **Co** | 0 | 7.13 | 11.93 | 15.35 | 22.69 | 29.79 | 37.22 | 46.32 | 42.58 |
| **Mn** | 0 | 6.35 | 12.46 | 14.13 | 21.11 | 28.66 | 38.52 | 48.02 | 45.79 |
| **Ni** | 0 | 8.26 | 10.26 | 14.35 | 23.06 | 29.16 | 37.22 | 46.15 | 44.5 |
| **Cd** | 0 | 6.39 | 11.26 | 15.9 | 22.39 | 28.76 | 38.66 | 47.87 | 41.8 |
|  | Removal % of *E. hormaechei* Wdcm scuf0000313 isolate by using 300% drainage concentration | | | | | | | | |
|  | **Befor R** | **12 h** | **24h** | **36 h** | **48 h** | **60 h** | **72 h** | **84 h** | **96 h** |
| **Zn** | 0 | 4.2 | 8.3 | 11.1 | 16.8 | 21.7 | 30.6 | 37.7 | 42.4 |
| **Fe** | 0 | 4.43 | 9.9 | 12.1 | 16.1 | 22.7 | 31.6 | 38.8 | 47.2 |
| **Pb** | 0 | 4.57 | 9.65 | 10.4 | 15.5 | 22.77 | 31.3 | 39.8 | 46.8 |
| **Co** | 0 | 3.4 | 9.36 | 12.4 | 17.8 | 20.9 | 29.7 | 36.6 | 45.6 |
| **Mn** | 0 | 3.49 | 9.4 | 10.3 | 16.1 | 22.2 | 31 | 36.4 | 43.6 |
| **Ni** | 0 | 5.75 | 8.99 | 12.9 | 15.8 | 21.1 | 31.4 | 38.3 | 46.4 |
| **Cd** | 0 | 5.9 | 8.96 | 12.2 | 15.9 | 19.5 | 29.6 | 38.5 | 40.5 |

**Table 4:** Consortium and solo application of bacteria against 100%, 200% and 300% of drainage concentration

|  | Removal % of three bacterial isolates by using 100% drainage concentration | | | | | | | | |
| --- | --- | --- | --- | --- | --- | --- | --- | --- | --- |
|  | **Befor R** | **12 h** | **24h** | **36 h** | **48 h** | **60 h** | **72 h** | **84 h** | **96 h** |
| **Zn** | 0 | 7.33 | 26 | 49.67 | 68.33 | 80.67 | 88.67 | 97.17 | 98.76 |
| **Fe** | 0 | 7.33 | 24.67 | 38 | 53.33 | 67 | 78.67 | 92.67 | 97.44 |
| **Pb** | 0 | 7.33 | 20 | 37.33 | 50.33 | 66.33 | 84.67 | 94.77 | 98.01 |
| **Co** | 0 | 7.33 | 17.33 | 31.33 | 46.33 | 51 | 69 | 77 | 80.31 |
| **Mn** | 0 | 7.33 | 18 | 30.33 | 41.33 | 51 | 62.33 | 74.33 | 77.62 |
| **Ni** | 0 | 7.33 | 27.67 | 39 | 50.33 | 60.33 | 70.67 | 83 | 92.42 |
| **Cd** | 0 | 7.33 | 26 | 41.33 | 54.67 | 64.33 | 79.67 | 91.67 | 95.07 |
| Removal % of three bacterial isolates by using 200% drainage concentration | | | | | | | | | |
|  | **Befor R** | **12 h** | **24h** | **36 h** | **48 h** | **60 h** | **72 h** | **84 h** | **96 h** |
| **Zn** | 0 | 6.57 | 15 | 22 | 29.33 | 38.17 | 44.33 | 52 | 61.19 |
| **Fe** | 0 | 6.93 | 15.27 | 21.33 | 28.83 | 36.67 | 44 | 51.33 | 57.57 |
| **Pb** | 0 | 6.3 | 14.4 | 20.93 | 28.63 | 36.8 | 43.87 | 50.67 | 68.50 |
| **Co** | 0 | 5.53 | 15 | 21.83 | 28.83 | 36.77 | 44.33 | 52.07 | 57.61 |
| **Mn** | 0 | 7.9 | 15.27 | 20.67 | 28.63 | 37.5 | 44.9 | 51.1 | 50.09 |
| **Ni** | 0 | 7 | 15 | 20.87 | 27.83 | 36.83 | 44.8 | 51 | 57.62 |
| **Cd** | 0 | 6.02 | 14.73 | 20.97 | 28.1 | 36.67 | 44.1 | 50.07 | 58.05 |
| Removal % of three bacterial isolates by using 300% drainage concentration | | | | | | | | | |
| **Befor R** | | **12 h** | **24h** | **36 h** | **48 h** | **60 h** | **72 h** | **84 h** | **96 h** |
| **Zn** | 0 | 6.11 | 9.55 | 12.78 | 20.17 | 25.91 | 33.69 | 42.12 | 43.53 |
| **Fe** | 0 | 6.12 | 10.83 | 14.02 | 19.79 | 26.74 | 34.42 | 41.78 | 46.05 |
| **Pb** | 0 | 6.82 | 11.13 | 14.02 | 20.88 | 27.91 | 35.97 | 44.39 | 48.12 |
| **Co** | 0 | 4.97 | 10.21 | 13.12 | 19.16 | 24.13 | 31.84 | 39.17 | 42.87 |
| **Mn** | 0 | 4.48 | 10.82 | 13.31 | 20.24 | 26.19 | 35.11 | 42.15 | 45.92 |
| **Ni** | 0 | 6.60 | 10.22 | 14.32 | 19.82 | 24.98 | 33.41 | 41.35 | 45.77 |
| **Cd** | 0 | 5.86 | 10.31 | 14.23 | 19.13 | 23.95 | 32.25 | 40.22 | 40.65 |
