# Supplementary material for: Bioremediation of industrial wastewater heavy metals using solo and consortium Enterobacter spp
Source: Environ Monit Assess. 2023 Oct 23;195(11):1357. doi: 10.1007/s10661-023-11951-x (PMC10593623; doi:10.1007/s10661-023-11951-x)
Supplement: Supplementary file 2 — (DOCX 924 kb) [file 10661_2023_11951_MOESM2_ESM.docx]

|  | **List of figures** |
| --- | --- |
| **Figure 1S-b** | The location of the industrial drainage area and its drainage point in Gulf of Suez proposing for bacterial isolation and industrial effluent treatment |
| **Figure 2S-b** | Microscopic examination of selected strains; (a) *E. Cloacae*, (b) *E. Kobei*, (c) *E. Hormaechei* |
| **Figure 3S-b** | Phylogenetic tree of molecular identified strains; (a) *E. Kobei*, (b) *E. Cloacae*, (c) *E. Hormaechei* |

| 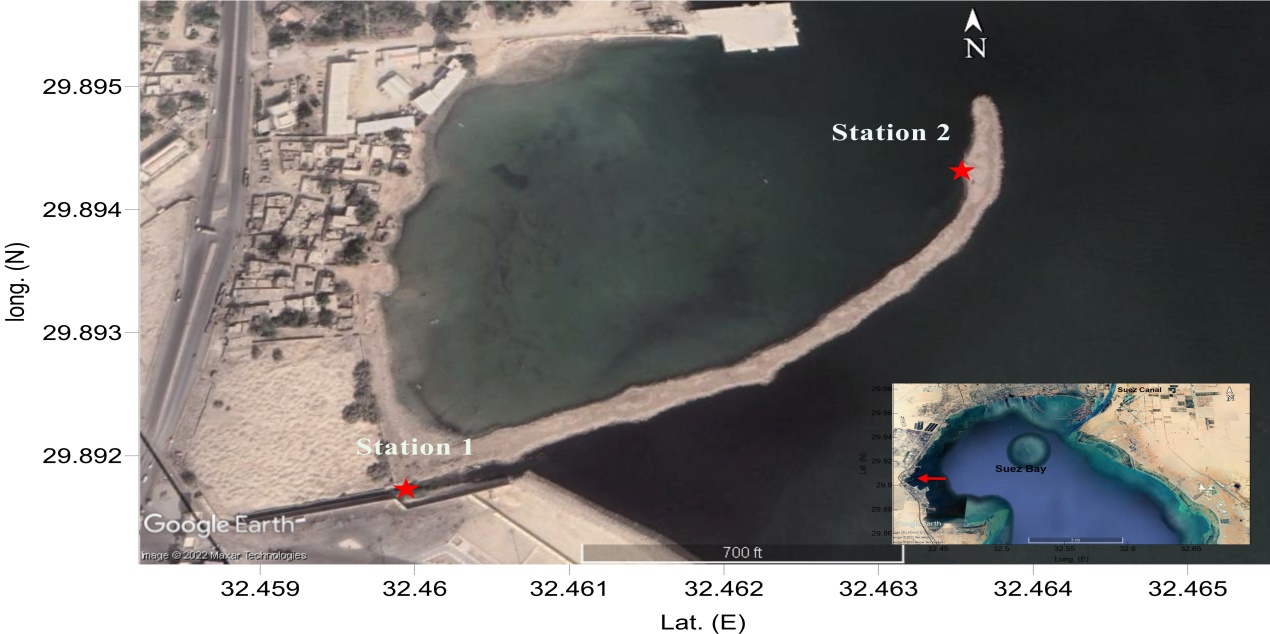 |
| --- |
| **Figure 1S-b**: The location of the industrial drainage area and its drainage point in Gulf of Suez proposing for bacterial isolation and industrial effluent treatment |

| (i-S4) | (ii-S5) | (iii-S7) |
| --- | --- | --- |
| 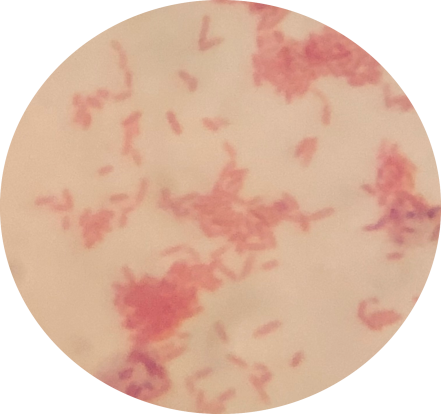 | 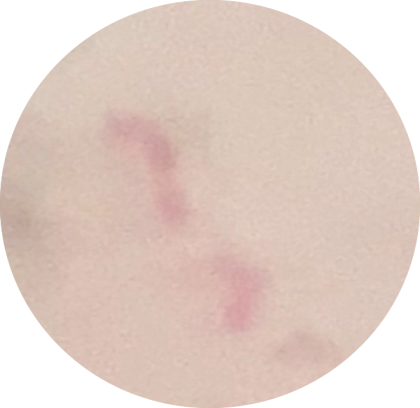 | 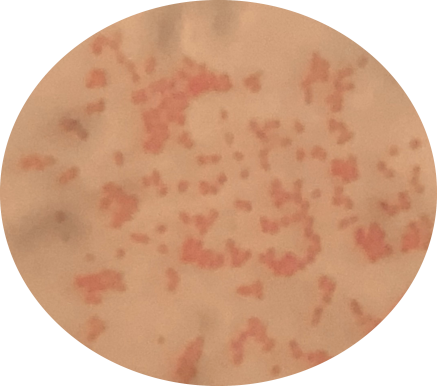 |
| **Figure 2S-b**: Microscopic examination of selected strains; (i) *E. Cloacae*, (ii) *E. Kobei*, (iii) *E. Hormaechei* | | |

| 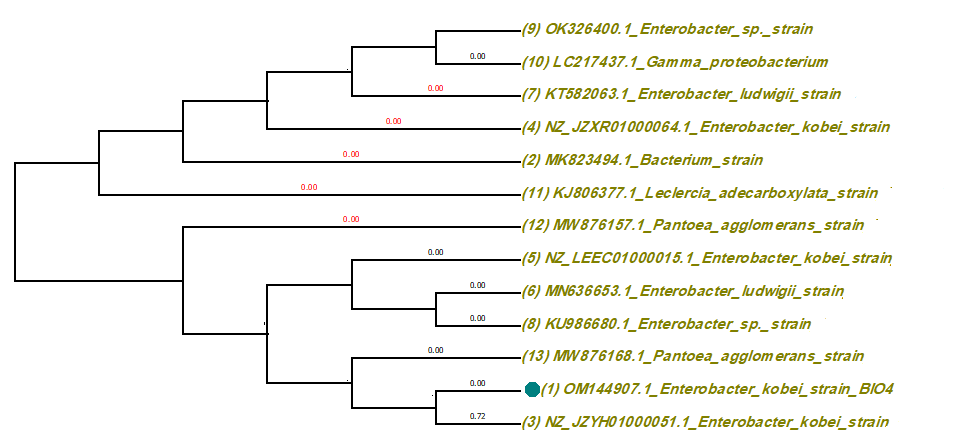  **i** | 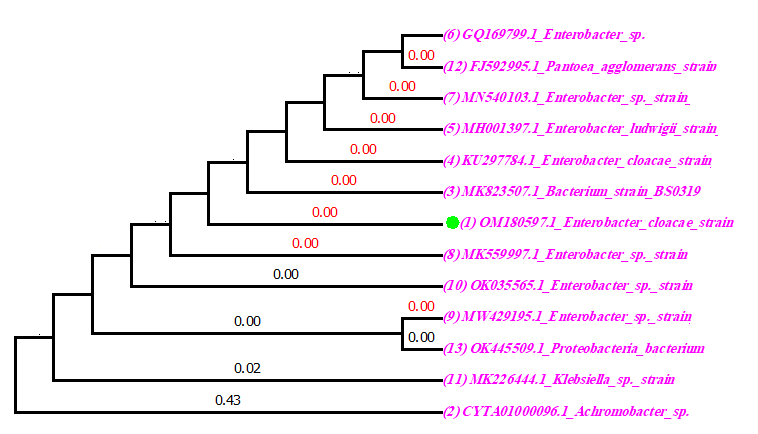  **ii** |
| --- | --- |
| 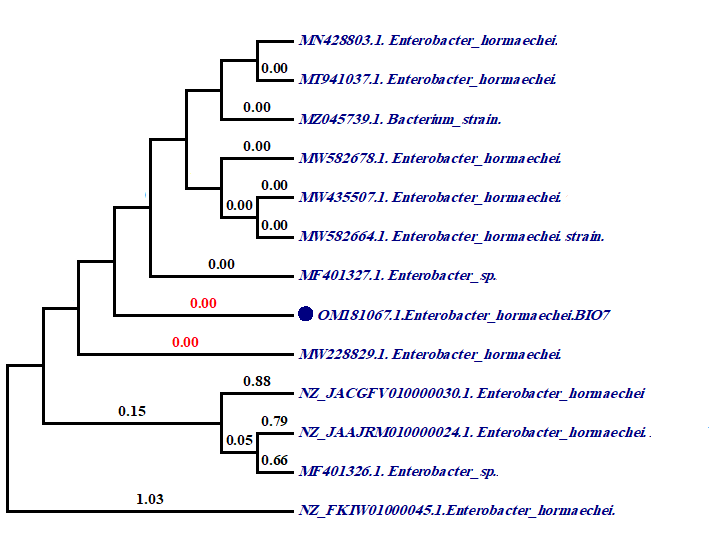  **iii** | |
| **Figure 3S-b:** Phylogenetic tree of molecular identified strains; (i) *E. Kobei*, (ii) *E. Cloacae*, (iii) *E. Hormaechei* | |
